# The efficacy and effectiveness of COVID-19 vaccines around the world: a mini-review and meta-analysis

**DOI:** 10.1186/s12941-023-00594-y

**Published:** 2023-05-19

**Authors:** Marzieh Soheili, Sorour Khateri, Farhad Moradpour, Pardis Mohammadzedeh, Mostafa Zareie, Seyede Maryam Mahdavi Mortazavi, Sima Manifar, Hamed Gilzad Kohan, Yousef Moradi

**Affiliations:** 1 Department of Pharmaceutical and Administrative Sciences, College of Pharmacy, Western New England University, 1215 Wilbraham Road, Springfield, MA 01119 USA; 2 Department of Physical Medicine and Rehabilitation, School of Medicine, Hamedan University of Medical Sciences, Hamedan, Iran; 3 Social Determinants of Health Research Center, Research Institute for Health Development, Kurdistan University of Medical Sciences, Sanandaj, Iran; 4 Department of Epidemiology and Biostatistics, School of Medicine, Kurdistan University of Medical Sciences, Sanandaj, Iran; 5 Pediatric Gastroenterology Fellowship, Department of Pediatrics, School of Medicine, Namazi teaching Hospital, Shiraz University of Medical Sciences, Shiraz, Iran; 6 Massachusetts College of Pharmacy and Health Sciences (MCPHS), 179 Longwood Avenue, Boston, MA 02115 USA; 7 Department of Pharmaceutical and Administrative Sciences, College of Pharmacy, Western New England University, 1215 Wilbraham Road, Springfield, MA 01119 USA

**Keywords:** COVID-19, SARS-CoV-2, Vaccine, Effectiveness, Efficacy

## Abstract

**Objectives:**

This meta-analysis evaluated the Efficacy and Effectiveness of several COVID-19 vaccines, including AstraZeneca, Pfizer, Moderna, Bharat, and Johnson & Johnson, to better estimate their immunogenicity, benefits, or side effects.

**Methods:**

Studies reporting the Efficacy and Effectiveness of COVID-19 vaccines from November 2020 to April 2022 were included. The pooled Effectiveness/Efficacy with a 95% confidence interval (95% CI) with Metaprop order was calculated. The results were presented in forest plots. Predefined subgroup analyses and sensitivity analyses were also performed.

**Results:**

A total of twenty articles were included in this meta-analysis. After the first dose of the vaccine, the total effectiveness of all COVID-19 vaccines in our study was 71% (95% CI 0.65, 0.78). The total effectiveness of vaccines after the second dose was 91% (95% CI 0.88, 0.94)). The total efficacy of vaccines after the first and second doses was 81% (95% CI 0.70, 0.91) and 71% (95% CI 0.62, 0.79), respectively. The effectiveness of the Moderna vaccine after the first and second dose was the highest among other studied vaccines ((74% (95% CI, 0.65, 0.83) and 93% (95% CI, 0.89, 0.97), respectively). The highest first dose overall effectiveness of the studied vaccines was against the Gamma variant (74% (95% CI, 0.73, 0.75)), and the highest effectiveness after the second dose was observed against the Beta variant (96% (95% CI, 0.96, 0.96)). The Efficacy for AstraZeneca and Pfizer vaccines after the first dose was 78% (95% CI, 0.62, 0.95) and 84% (95% CI, 0.77, 0.92), respectively. The second dose Efficacy for AstraZeneca, Pfizer, and Bharat was 67% (95% CI, 0.54, 0.80), 93% (95% CI, 0.85, 1.00), and 71% (95% CI, 0.61, 0.82), respectively. The overall efficacy of first and second dose vaccination against the Alfa variant was 84% (95% CI, 0.84, 0.84) and 77% (95% CI, 0.57, 0.97), respectively, the highest among other variants.

**Conclusion:**

mRNA-based vaccines against COVID-19 showed the highest total efficacy and effectiveness than other vaccines. In general, administering the second dose produced a more reliable response and higher effectiveness than a single dose.

## Introduction

The coronavirus disease 2019 (COVID-19) is an acute respiratory infection caused by the severe acute respiratory syndrome coronavirus 2 (SARS-CoV-2). This β-coronavirus is an enveloped, non-segmented positive-sense RNA virus, which primarily spreads through the respiratory tract [1–3]. COVID-19 infection is often associated with systemic inflammation and inflammatory biomarkers such as IL-6, IL-10, and TNF-α) increase in the patients [4–6]. Cough, fever, and shortness of breath are the dominant symptoms of COVID-19 infection. Additionally, fatigue, increased sputum production, sore throat, headache, and gastrointestinal symptoms might be observed [6–8]. Elderly patients with underlying disorders such as hypertension, chronic obstructive pulmonary disease, diabetes, and cardiovascular complications are more prone to develop acute respiratory distress syndrome. Other severe symptoms include septic shock, metabolic acidosis, and coagulation dysfunction, which might lead to death [9, 10]. Various medications have already been tested for treating COVID-19 patients. However, the evidence to support the beneficial effects of these drugs is often controversial [11–13]. Molnupiravir is the first oral antiviral drug that has recently shown a significant benefit in reducing hospitalization or death in COVID-19 patients [14].

According to the World Health Organization (WHO) report, from the emergence of COVID-19 in December 2019 to November 2021, more than 250,000,000 confirmed cases of COVID-19 have been reported, and more than five million deaths have been attributed to the disease globally [15]. Since the COVID-19 pandemic, several studies have started to develop safe and efficacious vaccines. Numerous clinical trials have been conducted to evaluate the efficacy and safety of experimental vaccines [16–18]. WHO reported as of November 8, 2021, more than seven billion vaccine doses have been administered worldwide [15]. Additionally, as per the WHO report, until November 9, 2021, 130 vaccine candidates were under clinical development, and 156 candidates were in the pre-clinical development phase. Different types of COVID-19 vaccines have been developed worldwide, including protein subunit, recombinant, viral vector, RNA- and DNA-based, and sub-unit vaccines [19].

Up to now, several COVID-19 vaccines have been authorized or approved for use. WHO issued an emergency use authorization for the Pfizer COVID-19 vaccine On December 31, 2020 (BNT162b2). Next, on February 15, 2021, the Astra-Zeneca/Oxford COVID-19 vaccine (manufactured by the Serum Institute of India and SKBio) received emergency use approval, followed by Ad26.COV2.S (developed by Janssen (Johnson & Johnson)) on March 12, 2021, and Moderna vaccine on April 30, 2021 [20]. Pfizer COVID-19 vaccine is a lipid nanoparticle formulation that contains a nucleoside-modified RNA against the S protein of the SARS-CoV-2 virus [21]. Moderna is a lipid nanoparticle–encapsulated nucleoside-modified messenger RNA vaccine encoding prefusion stabilized full-length spike protein of SARS-CoV-2 (24). The Oxford/AstraZeneca COVID-19 vaccine (ChAdOx1 nCoV-19 vaccine, AZD1222) contains a replication-deficient chimpanzee adenoviral vector ChAdOx1, delivering the SARS-CoV-2 structural surface glycoprotein antigen (spike protein; nCoV-19) gene (22, 23). Janssen is a non-replicating, recombinant human adenovirus type 26, containing a full-length SARS-CoV-2 S protein [22]. Bharat (CovaxinTM) is an inactivated-virus vaccine developed in Vero cells combined with Alhydroxiquim-II (Algel-IMDG), chemosorbed imidazoquinoline onto aluminum hydroxide gel. This complex is an adjuvant to boost immune response for longer-lasting immunity [23].

Careful planning for the COVID-19 vaccination program requires comprehensive review studies to evaluate the efficacy and safety of the vaccines. This study aims to conduct a meta-analysis to assess the Effectiveness and Efficacy of COVID-19 vaccines, including AstraZeneca, Pfizer, Moderna, Bharat, and Johnson & Johnson. Well-designed meta-analysis studies will provide a more accurate overview to evaluate Efficacy and safety outcomes compared to individual studies and contribute to a better understanding of the use of the vaccine in different populations.

## Materials and methods

The present systematic review and meta-analysis were conducted according to Preferred reporting items for systematic reviews and meta-analysis (PRISMA) guidelines for reviewing analytical observational studies [24].

### Search strategy and screening

International databases were searched to find all original published articles, including Medline (PubMed), Web of Science, Embase (Elsevier), Cochrane Library, Scopus, Ovid, and CINHAL, to retrieve all articles evaluating and reporting the efficacy and side effects of all COVID-19 vaccine (Pfizer–BioNTech, Oxford–AstraZeneca, Moderna, Janssen, CoronaVac, Covaxin, Novavax and Convidecia) in fully vaccinated and partially vaccinated people. The studies which have compared these items with non-vaccinated individuals were also included. In addition to searching the mentioned databases, gray literature was searched by reviewing articles in the first ten pages of Google scholar. A manual search was performed by reviewing references from related studies. This search was conducted with language limitations from November 2020 to September 2022. The search protocol was developed based on four primary roots involving “vaccination,“ “COVID-19,“ “Side effect,“ and “Efficacy.“ All related components to these keywords were “vaccinated”, “non-vaccinated”, “partial vaccinated”, “fully vaccinated”, “Pfizer–BioNTech”, “Oxford–AstraZeneca”, “Sinopharm BIBP”, “Moderna”, “Janssen”, “CoronaVac”, “Covaxin”, “Novavax”, “Convidecia”, “symptoms”, “signs” (“fever”, “cough”, “malaise”, “dyspnea”, “myalgia”, “sore throat”, and “diarrhea”), “thrombosis”, “emboli”, “thromboembolism”, “thromboembolic”, which were added to the searched queries based on scientific Mesh terms, EMTREE, and Thesaurus. Reference Manager bibliographic software was applied to manage searched citations. Duplicate entries were searched by considering the papers’ title, year of publication, authors, and specifications of types of sources. In case of questionable records, the texts were compared. After reviewing the primary search results, each article was double-checked by title and available abstract, and some of the articles were omitted based on the selection criteria. The evaluation of the considered papers was based on the inclusion and exclusion criteria by the two researchers separately (SM, MS). After the screening, (YM) selected the articles by evaluating their full texts.

### Eligibility criteria

We included all observational and interventional studies that assessed the Efficacy/Effectiveness and side effects of all types of COVID-19 vaccines (Pfizer–BioNTech, Oxford–AstraZeneca, Sinopharm BIBP, Moderna, Janssen, CoronaVac, Covaxin, Novavax and Convidecia) in fully vaccinated and partially vaccinated people. The studies comparing these items with non-vaccinated individuals were also included. We excluded duplicate citations, non-peer-reviewed articles in which the abstract and full text were unavailable, and other languages.

### Data extraction

After screening according to the three assessment steps for titles, abstracts, and full texts, the full text of each selected article was extracted for detailed analysis. The data were retrieved using a checklist recording author, publication year, type of study, mean age, sample size, number of positive tests, Effectiveness/Efficacy after one dose, Effectiveness/Efficacy after the second dose, and number of confirmed COVID cases, hospitalization, and death. From systematic search to final data extraction, all processes were followed independently by two research experts (PM, FM). After the screening, the data extraction was finally approved by (YM).

### Risk of bias

The qualitative evaluation of studies was done according to the Newcastle-Ottawa Quality Assessment Scale (NOS) [25] by two of the authors (FM, YM). This scale is designed to evaluate the qualitative properties of observational studies (random clinical trials, case-control, retrospective, cohort, and cross-sectional studies). NOS examined each study through six items in three groups: selection, comparability, and exposure. Stars were given to each item, and the maximum score was 9. If the scores assigned to the published articles differed, the external discussion method would be used [26, 27].

The Jadad checklist was used by two separate authors (PM and FM) to explore potential risks of bias in interventional studies. These scales include items to assess the adequacy of random sequence generation, allocation concealment, blinding, the detection of incomplete outcome data, selective outcome reporting, and other potential sources of bias [28].

### Statistical analysis

The random-effects model was used to calculate the pooled Effectiveness/Efficacy with a 95% confidence interval (95% CI) with Metaprop order. Calculating the cumulative relative risk (RR) with the 95% confidence interval and the meta set command was used considering the relative risk’s logarithm and logarithm standard deviation. Statistical analysis was performed using STATA 16.0 (Stata Corp, College Station, TX, USA), and statistical significance was considered at *P-Value* < 0.05. Heterogeneity among studies was evaluated by applying the I square value and reported as a percentage (%) to show the extent of variation between studies. A forest plot was used for presenting the meta-analysis results schematically. Egger’s test and funnel plot were applied to evaluate the publication bias. In addition, a subgroup analysis was done to identify different sources of heterogeneity.

## Results and discussion

### Characteristics of included studies and the participants

**Fig. 1:**
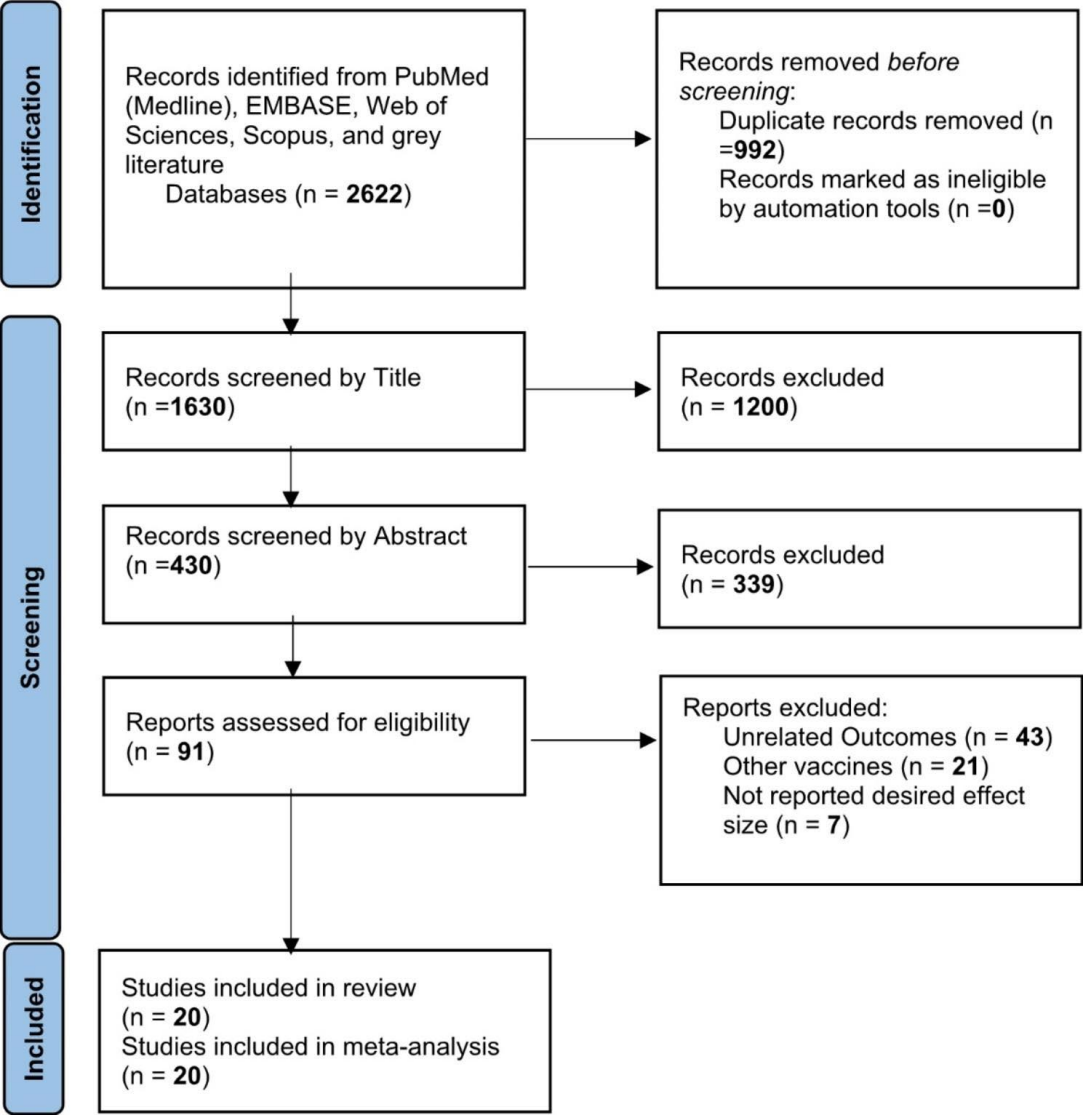
Identification of studies via databases and registers

A total of 2622 publications were screened for evaluating two items about COVID-19 vaccines: (I) Efficacy and (II) Effectiveness. These two items were assessed according to the virus variant (Alpha, Beta, Delta, and Gamma) and the type of vaccine (AstraZeneca, Pfizer, Moderna, Janssen, and Bharat). Data on other vaccines were not included due to inadequate published data. Of these publications, 20 studies met the systematic reviews’ inclusion criteria (non-randomized and randomized) and were included in our meta-analysis (Fig. 1).

One study was the cohort, four were randomized clinical trials (RCT), and fifteen were case-control. Clinical trials have evaluated vaccines’ efficacy, and observational studies such as cohorts and case controls have assessed their effectiveness. All selected papers were written in English. A total of 1,246,266 cases were included in this study that had received the COVID-19 vaccines. All vaccines were injected intramuscularly (IM). The participants were > 12 years old. The characteristics of included studies have been summarized in Table 1.

**Table 1:** The characteristics of included studies

Authors	Year	Method of Study	Sample Size (total)	Variant	vaccine code	Vaccine’s Type	Age	Efficacy After One Dose	Efficacy After Two Dose	1 d. Effectiveness against infection	2 d. Effectiveness against infection
J. Lopez Bernal et al.	2021	Case-Control	76,385	Alpha	1	Oxford-AstraZeneca		NR	NR	NR	NR
J. Lopez Bernal et al.	2021	Case-Control	62,484	Alpha	2	Pfizer-BioNTech		NR	NR	NR	NR
J. Lopez Bernal et al.	2021	Case-Control	18,061	Alpha	NR	Unvaccinated		NR	NR	NR	NR
J. Lopez Bernal et al.	2021	Case-Control	156,930	Alpha	NR	Total		NR	NR	NR	NR
K. R. W. Emary et al.	2021	RCT	8534	Alpha	1	Oxford-AstraZeneca		NR	0.6170	NR	NR
K. R. W. Emary et al.	2021	RCT	8534	non-Alpha	1	Oxford-AstraZeneca		NR	0.7730	NR	NR
S. A. Madhi et al.	2021	RCT	1467	Beta	1	Oxford-AstraZeneca		NR	0.1040	NR	NR
S. A. Madhi et al.	2021	RCT	1467	non-Beta	1	Oxford-AstraZeneca		0.7540		NR	NR
E. Pritchard et al.	2021	Cross- Sectional	41,018	Alpha	2	Pfizer-BioNTech		NR	NR	0.6600	0.8000
E. Pritchard et al.	2021	Cross-Sectional	41,018	Alpha	1	Oxford-AstraZeneca		NR	NR	0.6100	0.7900
E. Vasileiou et al.	2021	Cohort	620,154	NR	2	Pfizer-BioNTech		0.9100	NR	NR	NR
E. Vasileiou et al.	2021	Cohort	620,154	NR	1	Oxford-AstraZeneca		0.8800	NR	NR	NR
S. A. C. Clemens et al.	2021	RCT	9433	B.1.1.33	1	Oxford-AstraZeneca		NR	0.8820	NR	NR
S. A. C. Clemens et al.	2021	RCT	9433	Gamma	1	Oxford-AstraZeneca		NR	0.7260	NR	NR
S. A. C. Clemens et al.	2021	RCT	9433	Zeta	1	Oxford-AstraZeneca		NR	0.6870	NR	NR
S. A. C. Clemens et al.	2021	RCT	9433	Gamma	1	Oxford-AstraZeneca		NR	0.6360	NR	NR
S. A. C. Clemens et al.	2021	RCT	9433	Un determined	1	Oxford-AstraZeneca		NR	0.5660	NR	NR
A. R. Falsey et al.	2021	RCT	17,662	alpha and beta	1	Oxford-AstraZeneca		NR	0.7400	NR	NR
A. R. Falsey et al.	2021	RCT	8550	alpha and beta		Placebo		NR	0.7400	NR	NR
Singh C et al.	2021	Case-Control	1731	Delta	NR	Oxford-AstraZeneca, Bharat Biotech		NR	NR	0.5200	0.8300
Butt AA et al.	2021	Case-Control	108,720	Alpha	2	Pfizer-BioNTech		0.8400	0.9660	NR	NR
Desai D et al.	2021	Case-Control	2136	Delta	3	Bharat Biotech		NR	0.5700	NR	NR
Pilishvili TG et al.	2021	Case-Control	4931	NR	2	Pfizer-BioNTech		0.7760	0.8880	NR	NR
Pilishvili TG et al.	2021	Case-Control	4931	NR	1	Oxford-AstraZeneca		0.8890	0.9630	NR	NR
Thiruvengadam R et al.	2021	Case-Control	4360	Delta	1	Oxford-AstraZeneca		0.4620	0.6310	NR	NR
Li XN et al.	2021	Case-Control	277	Delta		SARS COV2		NR	0.7020	NR	NR
Young-Xu Y et al.	2021	Case-Control	75,546	Alpha, Epsilon. Iota	4	Moderna		NR	NR	0.6400	0.9500
Young-Xu Y et al.	2021	Case-Control	75,546	Alpha, Epsilon. Iota	2	Pfizer-BioNTech		NR	NR	NR	NR
Hitchings MD et al.	2021	Case-Control	61,360	Gamma	1	Oxford-AstraZeneca		NR	NR	NR	NR
Chemaitelly H et al.	2021	Case-Control	181,304	Alpha	1	Oxford-AstraZeneca		NR	NR	0.8810	1.0000
Chemaitelly H et al.	2021	Case-Control	181,304	Beta	1	Oxford-AstraZeneca		NR	NR	0.6130	0.9640
Chemaitelly H et al.	2021	Case-Control	181,304	total	1	Oxford-AstraZeneca		NR	NR	0.8160	0.9570
Pilishvili T et al.	2021	Case-Control	1845	NR	NR	Pfizer-BioNTech, Moderna		NR	NR	0.8170	0.9350
Gras-Valentí P et al.	2021	Case-Control	268	NR	2	Pfizer-BioNTech		NR	NR	0.5260	NR
Self WH et al.	2021	Case-Control	3689	NR	4	MODERNA		NR	NR	NR	NR
Self WH et al.	2021	Case-Control	3689	NR	2	Pfizer-BioNTech		NR	NR	NR	NR
Self WH et al.	2021	Case-Control	3689	NR	5	Janssen		NR	NR	NR	NR
Olson SM et al.	2021	Case-Control	464	NR	2	Pfizer-BioNTech		NR	NR	NR	0.9300
Bruxvoort KJ et al.	2021	Case-Control	5186	Delta	4	Moderna		NR	NR	0.7700	0.8670
Bruxvoort KJ et al.	2021	Case-Control	5186	Alpha	4	Moderna		NR	NR	0.9010	0.9840
Bruxvoort KJ et al.	2021	Case-Control	5186	Epsilon	4	Moderna		NR	NR	0.7630	0.9760
Bruxvoort KJ et al.	2021	Case-Control	5186	Gamma	4	Moderna		NR	NR	0.7420	0.9550
Bruxvoort KJ et al.	2021	Case-Control	5186	Iota	4	Moderna		NR	NR	0.8880	0.9570
Bruxvoort KJ et al.	2021	Case-Control	5186	Mu	4	Moderna		NR	NR	0.4580	0.9040
Bruxvoort KJ et al.	2021	Case-Control	5186	Other	4	Moderna		NR	NR	0.8430	0.9640
Bruxvoort KJ et al.	2021	Case-Control	5186	non-Delta	4	Moderna		NR	NR	NR	NR
Bruxvoort KJ et al.	2021	Case-Control	5186	Unidentified	4	Moderna		NR	NR	0.6760	0.7990
Bruxvoort KJ et al.	2021	Case-Control	5186	Total	4	Moderna		NR	NR	NR	NR
Ali K et al.	2021	RCT	3726	NR	1	Oxford-AstraZeneca		0.9300	1.0000	NR	NR
Ella R et al.	2021	RCT	16,973	All Varients	3	Bharat Biotech		NR	0.7080	NR	NR
Ella R et al.	2021	RCT	16,973	Delta	3	Bharat Biotech		NR	0.6520	NR	NR
Ella R et al.	2021	RCT	16,973	Kappa	3	Bharat Biotech		NR	0.9010	NR	NR
Ella R et al.	2021	RCT	16,973	Other	3	Bharat Biotech		NR	0.7300	NR	NR
Lutrick K et al.	2021	Cohort	243	Delta	2	Pfizer-BioNTech		NR	NR	NR	0.9200

### The overall effectiveness of COVID-19 vaccines

**Fig. 2:**
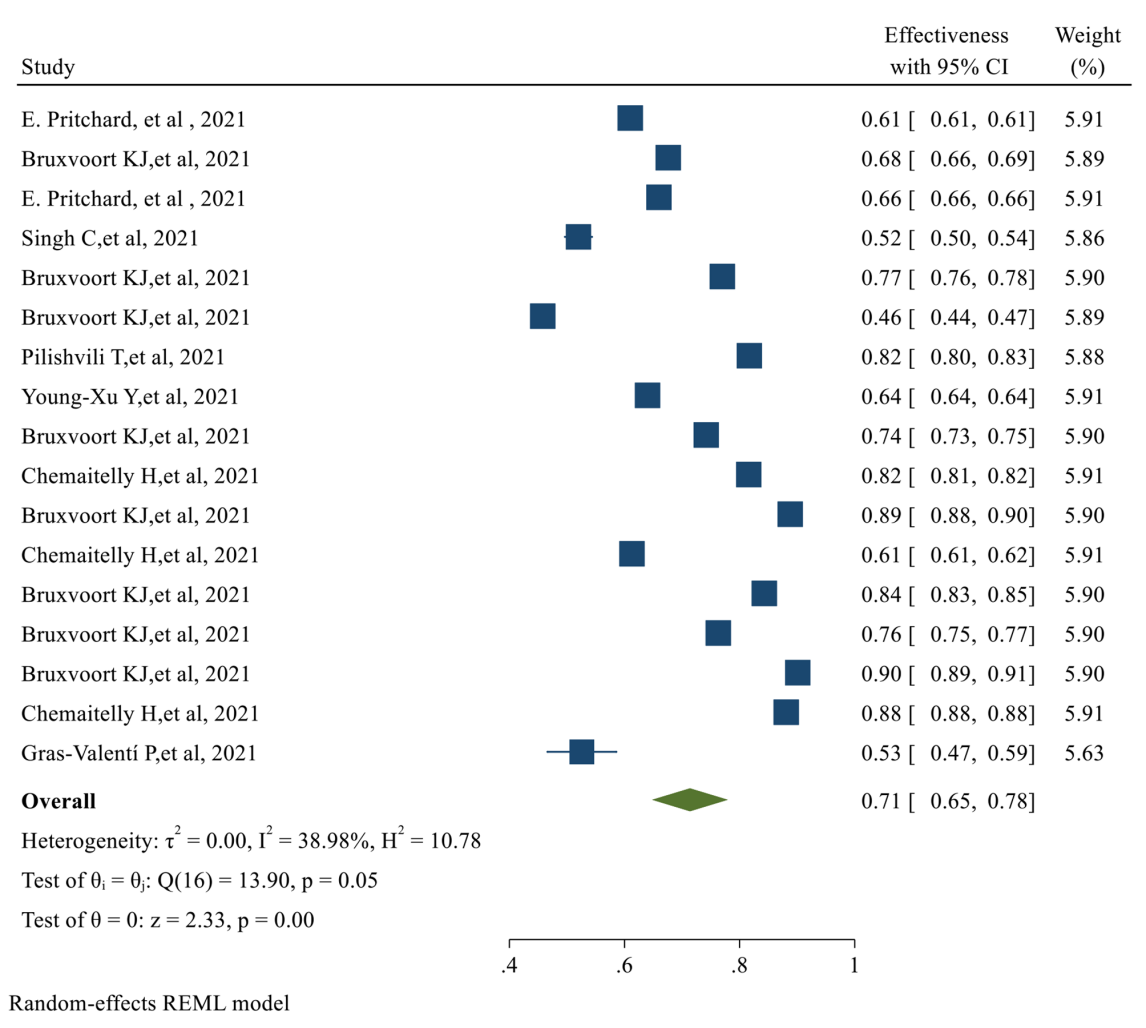
The overall Effectiveness of studied COVID-19 vaccines after the first dose The overall Effectiveness of vaccines after the second dose was 91% (95% CI 0.88, 0.94), with a significant *P-value* (*p-value < 0.05*) (Fig. 3)

After the first dose of the vaccine, the overall effectiveness of all COVID-19 vaccines was estimated to be 71% (95% CI 0.65, 0.78) (Fig. 2).

**Fig. 3:**
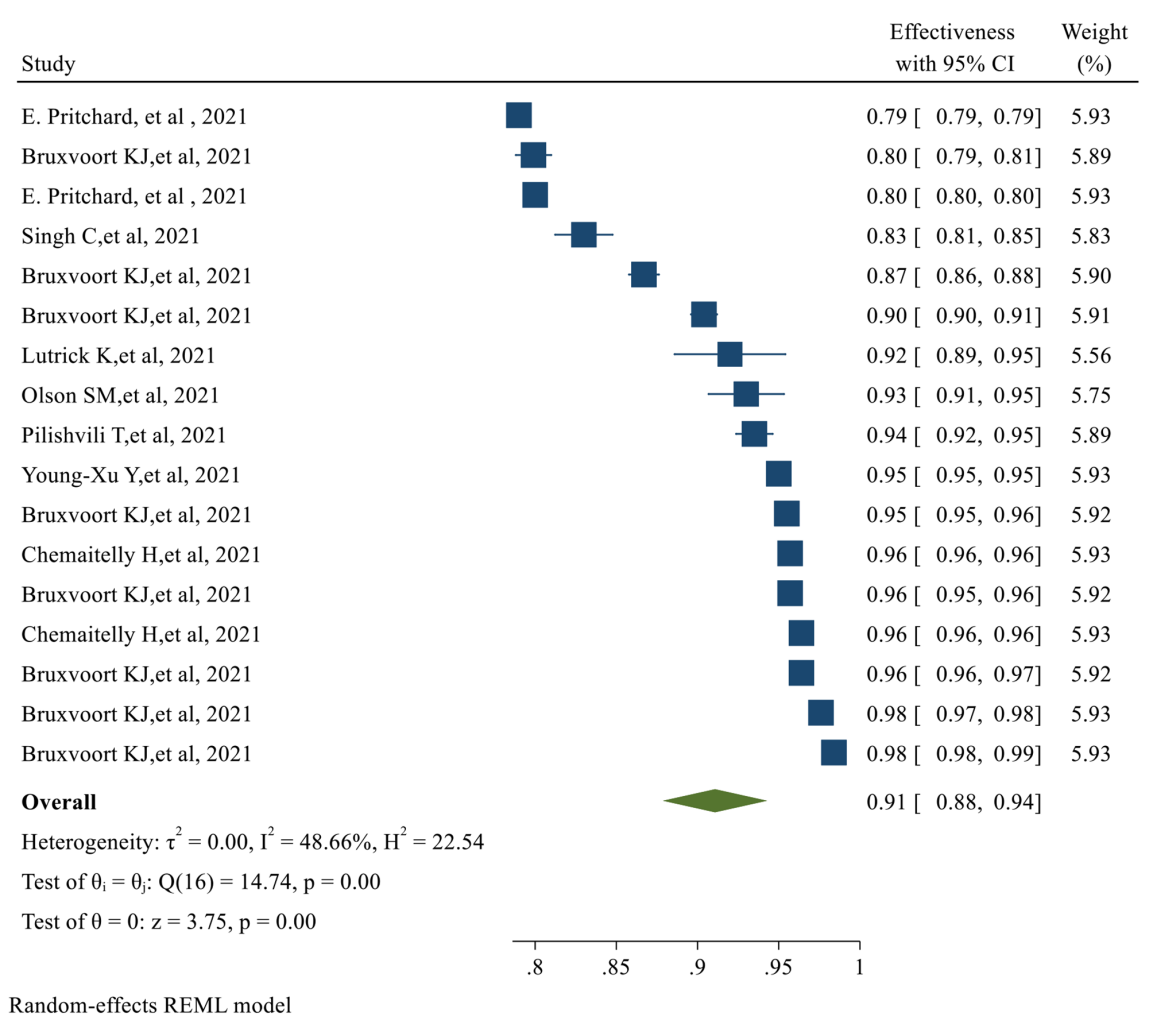
The overall Effectiveness of studied COVID-19 vaccines after the second dose. *The overall Efficacy of COVID-19 vaccines* The overall Efficacy of the first dose of the vaccines evaluated in our study was 81% (95% CI 0.70, 0.91) (Fig. 4)

**Fig. 4:**
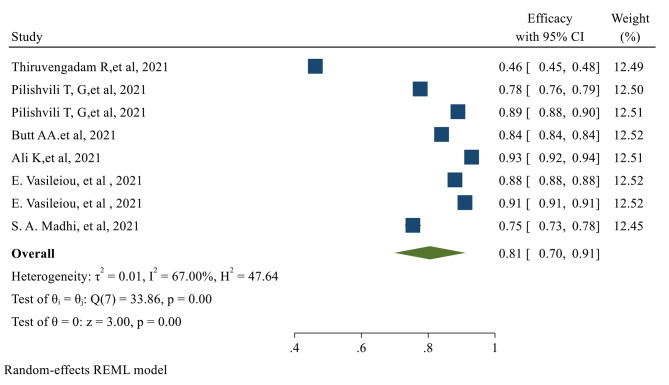
The overall Efficacy of the first dose of the studied vaccines After the second dose of vaccination, the overall Efficacy of vaccines was 71% (95% CI 0.62, 0.79) with a significant *P-value* (Fig. 5)

**Fig. 5:**
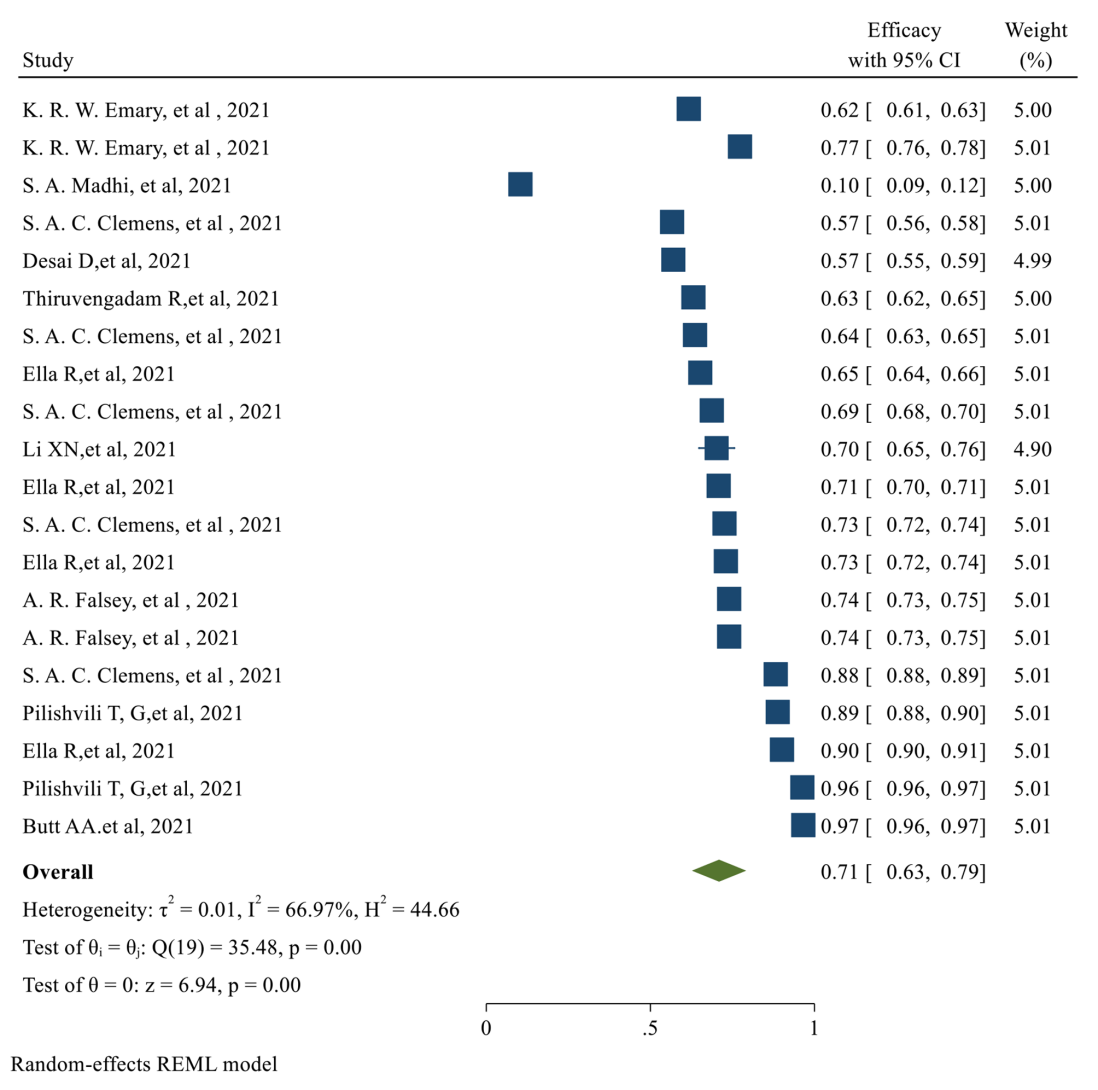
The overall Efficacy of the studied vaccines after the second dose

### The individual efficacy of COVID-19 vaccines

The efficacy after the first dose was evaluated only in 8 of the selected studies, which assessed the efficacy of the AstraZeneca and Pfizer vaccines. No data was published on the efficacy after the first dose for Moderna, Johnson & Johnson, and Bharat. After the first dose of AstraZeneca and Pfizer vaccines, the pooled efficacy was 78% (95% CI 0.062, 0.95) and 84% (95% CI 0.77, 0.92), respectively. Of the selected publications, eighteen studies reported the efficacy after the second dose of vaccinations. The published data for the second dose Efficacy was only available for AstraZeneca, Pfizer, and Bharat vaccines. The second dose pooled Efficacy for AstraZeneca, Pfizer, and Bharat was 67% (95% CI 0.54, 0.80), 93% (95% CI 0.85, 1.00), and 71% (95% CI 0.61, 0.82) respectively (Table 2).

**Table 2:** Vaccine Efficacy and Effectiveness by Vaccine type and variant

Efficacy or Effectiveness	Categories by vaccine or variant	No. Studies (Sample Size)	Pooled Efficacy	Between studies, heterogeneity assessment (%)	
				${\text{I}}^{2}$	P
1st Dose Efficacy	Astra	5 (634,638)	0.78 (0.62, 0.95)	77.09%	0.001
	Pfizer	3 (733,805)	0.84 (0.77, 0.92)	89.97%	0.001
2nd Dose Efficacy	Astra	11 (92,653)	0.67 (0.54, 0.80)	79.99%	0.001
	Pfizer	2 (113,651)	0.93 (0.85, 1.00)	-	-
	Baharat	5 (70,028)	0.71 (0.61, 0.82)	88.30%	0.001
	SARS COV2	1 (277)	0.70 (0.65, 0.76)	-	-
1st Dose Effectiveness	Moderna	9 (117,034)	0.74 (0.65, 0.83)	75.08%	0.043
	Astra	5 (586,661)	0.69 (0.55, 0.82)	89.00%	0.001
	Pfizer	3 (43,131)	0.67 (0.51, 0.83)	-	-
2nd Dose Effectiveness	Moderna	9 (117,034)	0.93 (0.89, 0.97)	73.40%	0.001
	Astra	4 (405,357)	0.89 (0.80, 0.97)	89.03%	0.001
	Pfizer	4 (43,570)	0.90 (0.83, 0.96)	44.32%	0.049
1st Dose Efficacy	Alpha	1 (108,720)	0.84 (0.84, 0.84)	-	-
	Delta	1 (4360)	0.46 (0.45, 0.48)	-	-
	Non-Beta	1 (1467)	0.75 (0.73, 0.78)	-	-
	NR	2 (9862)	0.83 (0.72, 0.94)		-
2nd Dose Efficacy	Alpha	3 (134,916)	0.77 (0.57, 0.97)	99.97%	-
	Beta	1 (1467)	0.10 (0.09, 0.12)	-	-
	Non-Alpha	1 (8534)	0.77 (0.76, 0.78)	-	-
	All Variants	1 (16,973)	0.71 (0.70, 0.71)	-	-
	Delta	4 (23,746)	0.64 (0.58, 0.69)	97.39%	-
	Gamma	2 (18,866)	0.68 (0.59, 0.77)	99.44%	-
	NR	2 (9862)	0.93 (0.85, 1.00)	99.51%	-
	Other	4 (52,812)	0.80 (0.69, 0.91)	99.91%	-
	Undetermined	1 (9433)	0.57 (0.56, 0.58)	-	-
1st Dose Effectiveness	Alpha	5 (344,072)	0.74 (0.62, 0.86)	99.98%	-
	Beta	1 (181,304)	0.61 (0.61, 0.62)	-	-
	Delta	2 (6917)	0.65 (0.40, 0.89)	99.71%	-
	Gamma	1 (5186)	0.74 (0.73, 0.75)	-	-
	Other	4 (20,744)	0.74 (0.55, 0.93)	99.92%	-
	Unidentified	1 (5186)	0.68 (0.66, 0.69)	-	-
	Total	1 (181,304)	0.82 (0.81, 0.82)	-	-
2nd Dose Effectiveness	Alpha	3 (87,222)	0.86 (0.73–0.98)	99.97%	-
	Beta	1 (181,304)	0.96 (0.96, 0.96)	-	-
	Delta	3 (7160)	0.87 (0.82, 0.92)	95.59%	-
	Gamma	1 (5186)	0.95 (0.95, 0.96)	-	-
	Other	4 (20,744)	0.95 (0.92, 0.98)	99.24%	-
	Unidentified	1 (5186)	0.80 (0.79, 0.81)	-	-
	Total	1 (181,304)	0.96 (0.96, 0.96)	-	-

### The individual effectiveness of COVID-19 vaccines

The first dose Effectiveness of the vaccines was evaluated in seventeen studies. For Moderna, AstraZeneca, and Pfizer, the pooled effectiveness after the first dose was 74% (95% CI 0.065, 0.83), 69% (95% CI 0.55, 0.82), and 67% (95% CI 0.51, 0.83) respectively. It was observed that the Effectiveness of Moderna after the first dose was higher than other types of vaccines. The second dose Effectiveness of the vaccines was reported in 17 studies. The pooled effectiveness after the second dose of Moderna, AstraZeneca, and Pfizer vaccines was 93% (95% CI 0.89, 0.97), 89% (0.80, 0.97), and 90% (95% CI 0.83, 0.96) respectively; Moderna had higher effectiveness after the second dose, among other studied vaccines (Table 2).

### Efficacy of the vaccines against the virus variants

The overall first and second-dose vaccination Efficacy against different COVID-19 variants is listed in Table 2. The first dose of overall vaccine Efficacy against the Alpha variant was 84%, which was higher than other variants (95% CI 0.84, 0.84). The overall efficacy of the first dose vaccination against the delta variant was only 46% (95% CI 0.45, 0.48), which was the lowest. Similarly, the highest second dose Efficacy was observed against the Alpha variant, which was 77% (95% CI 0.57, 0.97). The overall efficacy of the second dose against the Delta and Beta variants was 64% (95% CI 0.58, 0.69) and 10% (95% CI 0.09, 0.12), respectively.

### Effectiveness of the vaccines against the virus variants

The overall first and second-dose vaccination Effectiveness against different COVID-19 variants is reported in Table 2. The first dose Effectiveness of vaccination against the Gamma variant was 74% (95% CI 0.73, 0.75) which was more than other variants. However, the overall first dose Effectiveness was 82% (95% CI 0.81, 0.82). After the second dose, the highest effectiveness was against the Beta variant (96% (95% CI 0.96, 0.96)). The overall effectiveness after the second vaccination dose was 96% (95% CI 0.096, 0.96) (Table 2).

### The risk of confirmed COVID infection after vaccination (risk ratio)

Two categories of the selected studies assessed the risk ratio of COVID after vaccination: observational and experimental. Only the pooled risk ratio of AstraZeneca was evaluated in the experimental studies, which was 50% (95% CI 0.35, 0.71). In the observational studies, AstraZeneca and Moderna had the lowest pooled risk ratios, which were 18% (95% CI 0.04, 0.84) and 19% (95% CI 0.17, 0.22), respectively. Bharat had the highest pooled risk ratio (82% (95% CI 0.75, 0.89) (Table 3); however, the number of studies on the Bharat vaccine was fewer than other types of vaccines. Based on the reported experimental studies for the vaccine variants, the Beta variant had the highest (79% (95% CI 0.43, 1.44)), and the Gamma variant had the lowest risk ratio (31% (95% CI 0.18, 0.54)). In the observational studies, Delta had the highest (52% (95% CI 0.27, 1.01), and Gamma had the lowest risk ratio (2% (95% CI 0.02, 0.02)) (Table 3).

**Table 3:** The association of vaccination with the risk of confirmed COVID infection after vaccination (Risk Ratio)

Experimental and Observational	Categories by vaccine or variant	No. Studies (Sample Size)	Pooled Risk Ratio	Between studies, heterogeneity assessment (%)	
				${\text{I}}^{2}$	P
Total: Experimental	-	9 (71,487)	0.50 (0.35, 0.71)	79.68%	0.001
Total: Observational	-	17 (353,425)	0.28 (0.18, 0.45)	99.29%	0.001
Experimental by Vaccine	Astra	9 (142,974)	0.50 (0.35, 0.71)	79.68%	0.001
Observational by Vaccine	Baharat	1 (2136)	0.82 (0.75, 0.89)	-	-
	Janssen	1 (3689)	0.53 (0.41, 0.69)	-	-
	Moderna	2 (79,235)	0.19 (0.17, 0.22)	-	-
	Astra	4 (72,382)	0.18 (0.04, 0.84)	44.19%	0.074
	Pfizer	8 (195,706)	0.28 (0.18, 0.44)	77.02%	0.058
	SARS COV-2 vaccine	1 (277)	1.01 (0.64, 1.60)	-	-
Experimental by Variant	Alpha	1 (17,662)	0.44 (0.36, 0.53)	-	-
	Beta	1 (1467)	0.79 (0.43, 1.44)	-	-
	Gamma	2 (18,866)	0.31 (0.18, 0.54)	-	-
	Other	2 (18,866)	0.31 (0.22, 0.45)	-	-
	Un determined	1 (9433)	0.45 (0.27, 0.74)	-	-
	Non-Beta	1 (1467)	0.79 (0.43, 1.44)	-	-
Observational by Variant	Alpha	3 (259,812)	0.20 (0.17, 0.23)	76.62%	0.003
	Delta	5 (8747)	0.52 (0.27, 1.01)	63.89%	0.049
	Gamma	1 (61,360)	0.02 (0.02, 0.02)	-	-
	NR	2 (9862)	0.31 (0.18, 0.54)	-	-

## Discussion

Since the emergence of COVID-19, the effort to develop effective vaccines against the infection has been started. Due to the highly contagious nature of the virus, vaccination has been considered a significant measure in the fight against COVID-19. World Health Organization (WHO) allows countries to issue emergency use authorizations for COVID-19 vaccines in line with their national regulations and legislation. Domestic emergency use authorizations are issued at the countries’ discretion and are not subject to WHO approval. Up to now, several vaccines have been developed and marketed to limit the spread of COVID-19 infection. As of January 12, 2022, several COVID 19 vaccines have been given Emergency Use Listing (EUL), including those developed by Pfizer/BioNTech, AstraZeneca, Johnson & Johnson, Moderna, Sinopharm, Sinovac, Bharat Biotech, etc. [29].

Despite the significant role of COVID-19 vaccination in confining the infection, vaccines’ Efficacy and Effectiveness have not yet been comprehensively discussed. The present study meticulously looked into the Efficacy and Effectiveness of several vaccines.

Our analysis revealed that the overall effectiveness of the studied vaccines after the first dose is significantly less than their effectiveness after the second dose. The first dose’s effectiveness was evaluated in 17 studies. After the first dose, Moderna, AstraZeneca, and Pfizer’s Effectiveness was 74%, 69%, and 67%, respectively. The Effectiveness of Moderna after the first dose was higher than other types of studied vaccines. Second dose Effectiveness was evaluated in 17 studies. After the second dose of Moderna, AstraZeneca, and Pfizer vaccination, the effectiveness was 93%, 89%, and 90, respectively. Moderna provided higher effectiveness after the second dose among other studied vaccines. Therefore, administering the second dose should produce a more reliable response and higher effectiveness than a single dose.

Surprisingly, the overall efficacy of the first dose was significantly more than the second dose; 81% (95% CI 0.70, 0.91) for the first dose compared to 71% (95% CI 0.62, 0.79) for the second dose. This can be explained by the fact that the efficacy after the first dose was evaluated only in 8 studies that assessed only AstraZeneca and Pfizer vaccines. No data was available regarding the efficacy after the first dose of Moderna, Bharat, and Johnson & Johnson vaccines. We observed that the first dose Efficacy of the Pfizer vaccine is significantly more than the AstraZeneca vaccine. The Efficacy for AstraZeneca and Pfizer after the first dose vaccination was 78% and 84%, respectively. Concerning the second dose Efficacy, the published data were available only for AstraZeneca, Pfizer, and Bharat. In Total, eighteen studies evaluated the efficacy of these vaccines after the second dose. The Efficacy for AstraZeneca, Pfizer, and Bharat was 67%, 93%, and 71%, respectively.

We also investigated the Efficacy and Effectiveness of the first and second-dose vaccination against the COVID-19 virus variants. The overall efficacy of vaccination against the Alfa variant after the first dose was 84%, which was more than other variants. The highest efficacy after the second dose vaccination was also observed for the Alpha variant (77%). The first dose’s effectiveness against the Gamma variant was the highest (74%). Although, the overall first dose effectiveness was 82%. The highest second dose Effectiveness was against the Beta variant (96%), and the overall effectiveness after the second vaccination dose was 96% against all variants.

Up to now, there are other meta-analyses published on the efficacy and effectiveness of the COVID-19 vaccines. For example, in the meta-analysis reported by Pormohammad et., al, the efficacy of mRNA-based and adenovirus-vectored COVID-19 vaccines in phase II/III randomized clinical trial has been reported as 94.6% (95% CI 0.936–0.954) and 80.2% (95% CI 0.56–0.93), respectively. Additionally, the mRNA-based vaccines showed the highest reported side effects except for diarrhea and arthralgia [30]. However, the research had not reported the efficacy against different variants of the COVID-19 virus. Moreover, the Efficacy and Effectiveness of individual vaccines have not been mentioned; the vaccine Efficacy has been reported based on the vaccine classes. Another meta-analysis reported that the effectiveness of the Pfizer-BioNTech and Moderna vaccines was 91.2% and 98.1%, respectively, while the effectiveness of the CoronaVac vaccine was 65.7% in fully vaccinated individuals [31]. However, this study has not reported the effectiveness of the vaccines against COVID-19 variants or their efficacy.

Additionally, A previously reported network meta-analysis of various COVID-19 vaccines found Moderna was the most effective vaccine against COVID-19 infection, with an efficacy rate of 88%, followed by Sinopharm and Bharat. The least effective vaccines were Coronavac, Curevac, and AstraZeneca. The mRNA-based vaccines were superior in preventing infection and symptomatic infection, while the inactivated vaccines were most effective in preventing severe COVID-19 infection. Concerning safety, Sinopharm had the highest safety profile in local side effects, while ZF2001 had the highest safety in unsolicited side effects. Inactivated vaccines had the best safety profile in local and systemic side effects, while mRNA-based vaccines had the poorest safety profile. Thromboembolic events were reported after J&J, AstraZeneca, Pfizer, and Moderna vaccine administration. However, no confirmed vaccine-Induced Thrombotic Thrombocytopenia (VITT) cases were reported after mRNA vaccines [32].

It is necessary to mention that some vaccines’ overall or variant-specific Effectiveness and Efficacy are unavailable after the first or second dose. Moreover, the timing of the second dosing of the vaccines is not elicited in some trials, which may have led to the lower observed overall efficacy after the second dose. Additionally, some reports had noticeable bias by not including enough samples or not considering a broad enough geographical, economic, and age diversity.

We searched various databases and websites to include the maximum number of relevant publications to prevent database bias; after performing Egger’s regression test, we did not find significant publication bias. However, publication bias and heterogeneity for some pooled results must be considered when interpreting the outcomes.

Despite the valuable information provided by this meta-analysis, the study has some limitations to consider, such as the time frame of the studies (November 2020 to April 2022), the exclusion of unpublished data or ongoing investigations, the subjectivity of study selection criteria, and the limited number of vaccines evaluated. Additionally, the study did not consider differences in vaccine distribution among countries or provide data on the vaccines’ effectiveness against severe disease, hospitalization, or death. Despite its limitations, the meta-analysis highlights the need to continue monitoring the vaccines’ effectiveness.

## Conclusion

In conclusion, Moderna, an mRNA-based vaccine, showed the highest total effectiveness after the first dose. Although the Pfizer vaccine showed a higher Efficacy after the first and second doses than AstraZeneca and Bharat, our conclusion has some limitations due to the lack of any published study regarding the Moderna and Johnson & Johnson vaccines’ efficacy. First-dose vaccination generally showed the highest overall effectiveness against the Gamma variant. Second dose vaccination showed a 96% overall Effectiveness against all variants. The efficacy of vaccination against the Alfa variant after the first dose was more than other variants. The highest efficacy after the second vaccination dose was also observed for the Alpha variant. Due to the timeline of the studies, all the vaccines are missing longer-term Efficacy and Effectiveness evaluations. This meta-analysis incorporated all relevant studies for summarizing and analyzing the Effectiveness and Efficacy of several vaccines for COVID-19. The results of this study support the overall Efficacy and Effectiveness of all studied COVID-19 vaccines and support the ongoing global public health effort for vaccination against COVID-19.

## Data Availability

The data extracted for analyses are available by the corresponding author upon reasonable requests.

## Abbreviations

COVID-19: Coronavirus Disease 2019
SARS-CoV-2: Severe Acute Respiratory Syndrome Coronavirus 2
WHO: World Health Organization
